# Comparative Analysis of Intestine Microbiota of Four Wild Waterbird Species

**DOI:** 10.3389/fmicb.2019.01911

**Published:** 2019-08-20

**Authors:** Sivan Laviad-Shitrit, Ido Izhaki, Maya Lalzar, Malka Halpern

**Affiliations:** ^1^Department of Evolutionary and Environmental Biology, Faculty of Natural Sciences, University of Haifa, Haifa, Israel; ^2^Bioinformatics Service Unit, University of Haifa, Haifa, Israel; ^3^Department of Biology and Environment, Faculty of Natural Sciences, University of Haifa at Oranim, Tivon, Israel

**Keywords:** phylosymbiosis, waterbird, eco-evolution, microbiota, microbiome, bacterial community composition

## Abstract

Waterbirds are ubiquitous and globally distributed. Yet, studies on wild waterbirds’ gut microbiota are still rare. Our aim was to explore and compare the gut microbial community composition of wild waterbird species. Four wild waterbird species that are either wintering or all-year residents in Israel were studied: great cormorants, little egrets, black-crowned night herons and black-headed gulls. For each bird, three intestinal sections were sampled; anterior, middle and posterior. No significant differences were found among the microbiota compositions in the three intestine sections of each individual bird. Each waterbird species had a unique microbial composition. The gut microbiota of the black-headed gulls’ fundamentally deviated from that of the other bird species, probably due to a very high abundance (58.8%) of the genus *Catellicoccus* (*Firmicutes*). Our results suggest a correlation between the waterbird species’ phylogeny and their intestine microbial community hierarchical tree, which evinced phylosymbiosis. This recent coinage stands for eco-evolutionary patterns between the host phylogeny and its microbiota composition. We conclude that eco-evolutionary processes termed phylosymbiosis may occur between wild waterbird species and their gut microbial community composition.

## Introduction

All living organisms host microorganism assemblages that are referred to as microbiomes. The gut microbiota of an organism is established immediately after birth and changes due to the host life-style, diet, environmental conditions, genome, etc. (Nicholson et al., 2012; Sommer and Bäckhed, 2013). Gut microbiota is considered to affect the host physiology, nutritional status, and its behavior under stress conditions (Sekirov et al., 2010). Rosenberg and Zilber-Rosenberg (2013) suggested that an organism should be defined together with its endogenous microorganisms as a holobiont. Moreover, the host together with its endogenous microbial community constantly undergoes a mutual evolutionary process and thus, the hosts’ genome and its microbiome can be referred to as a “hologenome.” Shapira (2016) distinguished between a host-adapted core microbiota that may be vertically transmitted and a transient microbiota that their pool may depend on environmental conditions. The evolutionary relation between the host and its microbiome was redefined by Theis et al. (2016) who suggested that the host and its microbiome undergo eco-evolutionary processes. These processes between a host’s and its microbiota is termed phylosymbiosis (Brucker and Bordenstein, 2012a, b; Theis et al., 2016). Phylosymbiosis means that a host’s microbial community composition is selected so as to maintain the host’s evolution, a process that is not random (Richardson, 2017). Phylosymbiosis does not depend on vertical transmission, co-evolution or co-diversification. Namely, the microbial community composition in a host may be assembled with each new generation (Brooks et al., 2016).

Birds are ubiquitous and globally distributed. Hird (2017) pointed out that to understand birds’ evolutionary biology we need to study their microbiomes. They fly, therefore change their environment frequently and this may have a direct effect on their microbiomes (Hird, 2017). As far as we know, studies on wild waterbird gut microbiota are at present relatively very rare (Grond et al., 2018). The few that have been conducted on wild waterbirds, did not sample the gut contents but the birds’ feces (Zhao et al., 2017; Hird et al., 2018). Nevertheless, other studies that were conducted on domestic waterfowl, also examined feces and not intestine content (Xu et al., 2017).

The gastrointestinal tract of birds is relatively shorter compared to mammalians. The digesting process takes less than 3.5 h which leads to a very selective and adjusted microbiome. It consists of esophagus, crop, proventriculus, gizzard, small intestines (duodenum, jejunum, and ileum), cecum, colon, and cloaca (Pan and Yu, 2013). Bodawatta et al. (2018) found differences in the microbiota along the digestive tract of New Guinean passerine bird species. These differences were correlated to the birds’ diet (insectivores or omnivores birds). In contrast, in a study on wild red-billed choughs (*Pyrrhocorax pyrrhocorax*), no significant differences were found between the relative abundances of the four dominant phyla in the oropharynx, gizzard, small intestine, and large intestine. Also, no significant differences were found in diversity and richness indices of these four gastrointestinal locations (Wang et al., 2019).

Migratory birds can serve as vectors and disseminators of different bacterial species including pathogens. Using cloning methods, Ryu et al. (2014) showed that migratory shorebirds are potential reservoirs of pathogenic *Campylobacter* species. Halpern et al. (2008) suggested that migratory waterbirds are able to disseminate *Vibrio cholerae*, and explained the dispersal in that some waterbird species feed on copepods, chironomids and/or fish, all of which are reservoirs of the cholera bacterium (Laviad-Shitrit et al., 2019). Halpern and Senderovich (2015) suggested a novel concept regarding the predator–prey scenario. The prey with its microbiome is consumed by a predator. As a result, some of the prey’s microbiome may colonize the predator’s gut, proliferate and become an integral part of the predator’s gut microbiota. The rest of it will probably simply pass through the predator’s gut and be expelled from its intestinal tract. Recently we demonstrated that great cormorants became infected with *V. cholerae* through their tilapia (fish) prey (Laviad-Shitrit et al., 2017).

Israel is located in the Middle East at the intersection of three continents (Asia, Europe and Africa) and serves as an important geographical “bottleneck” for migratory waterbirds but also inhabits many waterbird species that are wintering or all-year residents. About one million soaring waterbird species pass through Israel every year in the fall (Shamoun-Baranes et al., 2006). This makes this country a suitable place to sample and study waterbird microbiomes.

In the current study we addressed the following specific hypotheses: (1) There are greater similarities between the microbiota composition of various parts of the intestine of the same individual compared to the microbiota composition of other individuals of the same host species, (2) the microbial communities of closely related waterbird species will be more similar than less related species, and (3) the microbial communities of individuals of the same host species will be much more similar than of individuals of other species. To test these hypotheses we explored the gut microbial community composition of four wild waterbird species: great cormorants, little egrets, black-crowned night herons and black-headed gulls. Our results showed that the microbial assemblage similarity among individuals of the same host species was more distinct than the similarity among assemblages that harbor the three intestine parts within each individual. Our results also demonstrated that each waterbird species has a unique microbiota assemblage and that there is a correlation between the waterbird species’ phylogeny and their gut microbial community composition.

## Materials and Methods

### Ethics Statement

Fish ponds are known as a habitat for many waterbird flocks because they provide the birds a variety of food. These flocks can destroy entire fish ponds by eating the fish, causing losses amounting to millions of dollars. Accordingly, fishermen in Israel are allowed to hunt a fixed number of waterbird individuals belonging to three different species: great cormorant (*Phalacrocorax carbo*), little egret (*Egretta garzetta*) and black-crowned night heron (*Nycticorax nycticorax*). All EU states, as well as Israel, belong to the Agreement on the Conservation of African-Eurasian Migratory Waterbirds (AEWA)^1^ under the Convention on Migratory Species (CMS) of the United Nations Environmental Program (UNEP). In Israel, the license to hunt and shoot waterbirds as a mean of controlling them in fish ponds abides by the regulations for wild animals’ protection of 1955 and 1976 [regulation no. 5A(2-4), 1976; in Hebrew]. Black-headed gulls (*Chroicocephalus ridibundus*) were collected (only 5 individuals) after they had been struck by stray bullets fired by bird hunters.

### Bird Samplings

Four different wild waterbird species were collected near fish ponds between January and August 2014: (i) little egrets (*n* = 11), (ii) black-crowned night herons (*n* = 8), (iii) great cormorants (*n* = 7), and (iv) black-headed gulls (*n* = 5). Little egrets, black-crowned night herons and the great cormorants were collected at Ma’agan Michael (32°33′31.6^″^N 34°54′37.6^″^E). Black-headed gulls were collected in Beit Shean valley (32°29′05.4^″^N 35°31′45.9^″^E).

The two species of herons (little egret and black-crowned night herons) are all-year residents that breed in mixed colonies in Israel. These two species are also very common transients and wintering species. We collected 7 egrets and 2 herons in winter and 4 and 6, respectively, in summer. The birds sampled in summer are all-year residents but those in winter might be either migrants or winterings. The great cormorant and the black-headed gulls are common wintering species in Israel. Capture of ringed birds showed that many of the cormorants arrive to Israel from Ukraine whereas the gulls arrive from central Europe and Russia (The Israel Ornithological Center, Unpublished data).

All birds were taken directly to the lab, where three parts of the intestine were sampled with sterile needles: (A) the beginning of the intestine (hereinafter anterior), (B) the middle of the intestine (hereinafter middle), and (C) close to the intestine end (hereinafter posterior). The samples were transferred to 2 ml sterile tubes that contained 0.5 ml absolute ethanol. The tubes were kept at –20°C until DNA had been extracted.

### DNA Extraction From Intestine Samples

Tubes were centrifuged for 30 min at maximum speed and the ethanol residues were removed with a sterile tip. DNA was extracted from the birds’ gut samples with a DNA isolation kit (DNeasy Blood and Tissue, Qiagen, Germany) according to the manufacturer’s instructions and with minor modifications as described previously (Laviad-Shitrit et al., 2017). The extracted DNA was kept at –20°C.

### Generation of the 16S rRNA Gene Library

The V4 variable region of the 16S rRNA gene was PCR-amplified from the extracted DNA using the primer pair CS1_515F (ACACTGACGACATGGTTCTACAGTGCCAGCMGCCGCGG TAA) and CS2_806R (TACGGTAGCAGAGACTTGGTCTGGAC TACHVGGGTWTCTAAT) (synthesized by Sigma Aldrich, Israel) as described previously (Caporaso et al., 2012).

Amplification reactions were performed in a volume of 25 μl with the EmeraldAmp MAX HS PCR Master Mix (Takara Bio Inc., Otsu, Shiga, Japan). The primers’ concentrations were 0.5 ng/μl, and 10–100 ng genomic DNA was added to each PCR reaction tube. PCR conditions were 95°C for 5 min, followed by 28 cycles of 30 s at 95°C; 45 s at 55°C and 30 s at 68°C, and a final elongation step of 7 min at 68°C. PCR products were verified to contain amplification by agarose gel electrophoresis. Controls that did not contain DNA templates were PCR amplified and checked for potential contaminations. No contamination was found.

### Illumina MiSeq Sequencing

Illumina MiSeq sequencing was performed at the DNA Services (DNAS) facility – University of Illinois in Chicago (UIC). The sequencing protocol has been described previously (Aizenberg-gershtein et al., 2015). In brief, all samples were amplified in a second PCR amplification with a distinct primer pair for each sample that contained a unique 10-base barcode, obtained from the Access Array Barcode Library for Illumina (Fluidigm, South San Francisco, CA; Item# 100-4876). Then, pooled diluted libraries were sequenced with Illumina MiSeq 600-cycle sequencing kit version 3, and analyzed with Casava 1.8 (pipeline 1.8). The nucleotide reads length were 200 (paired end, 2 × 200). PhiX DNA was used as a spike-in control. Barcode sequences from Fluidigm were provided to the MiSeq server, and sequences were automatically binned according to 10-base multiplex identifier (MID) sequences. Finally, raw reads were recovered as FASTQ files and are available at the NCBI^2^ database under BioProject accession number PRJNA336254.

### Data Analysis

Bioinformatics analysis was performed with MOTHUR v. 1.37.4. The operational taxonomic unit (OTU)-based approach of the MiSeq Standard Operating Procedure (SOP) was followed (Kozich et al., 2013). All reads were paired using the make.contigs command. Then, all sequences with ambiguities or with homopolymers that were longer than 8 bases were removed. Sequences were trimmed to a unified length of 292 base pairs. Sequences were aligned using the SILVA-compatible alignment database available in MOTHUR.

Chimeric sequences were removed by UCHIME (Edgar et al., 2011) and any non-bacterial (unknown, chloroplast, mitochondrial, archaeal and eukaryotic) sequences were also removed using the remove.lineage command. The filtered set of sequences were then clustered into OTUs at 97% sequence similarity threshold to the MOTHUR cluster.split command. Then OTU taxonomy was determined by the MOTHUR classify.otu command and the SILVA non-redundant small subunit rRNA database (version 128). Consensus sequences for each OTU were calculated by MOTHUR consensus.seqs command. Next, the entire dataset was randomly subsampled to 20,000 sequences per sample. Where required, we merged the section samples of each individual waterbird. Since not all individuals rendered samples from all the intestine sections, the average read counts were calculated and used for further analysis.

### Statistical Analysis

To assess the effects of waterbird species and intestinal section on the composition of the sampled microbiota and their interactions, we performed the ADONIS test using the Bray–Curtis dissimilarities between microbiota compositions and Unifrac analysis. We tested the model of waterbird species, intestinal section and individual bird with all interactions (with 10,000 permutations). ADONIS was implemented in the R package vegan (Oksanen et al., 2015) and Unifrac was implemented in MOTHUR program.

For the most dominant phyla we performed One-way ANOVA to study the differences in bacterial abundances among the bird species and at each of the gut sections (anterior, middle and posterior). We used Bonferroni multiple comparison test to evaluate the significance of the differences among species and among intenstine parts of each phyla.

#### α-Diversity

To assess the microbial diversity of the waterbird intestines’ bacterial communities, we performed rarefaction analysis using R package iNEXT (Hsieh et al., 2016). We likewise calculated the observed and estimated (Chao1) richness, the dominance (1- Simpson index) and evenness for both OTUs and genus levels of classification. All indices were calculated with PAST software (Hammer et al., 2001). We applied the Kruskal–Wallis test to compare the species for alpha-diversity parameters. Where required, *post hoc* multiple comparisons were performed by the Dunn test.

#### β-Diversity

Similarity of the gut microbiota of the different species and of the different individuals in a specific species, was detected by a non-metric multidimensional scaling analysis (nMDS), based on the Bray–Curtis similarity matrix with Primer v7 software. The bacterial community profile was tested by analysis of similarity (ANOSIM) on all species (*p* < 0.05), with *post hoc* pair-wise comparisons and Bonferroni corrections (*p* < 0.05), with *R*.

To present a Venn diagram (to show the unique and shared OTUs of the waterbird species and of the different gut sections of each species) β*-diversity* was analyzed by the MOTHUR program (version v. 1.37.4.) as described previously (Kozich et al., 2013).

#### Indicator Genera

To find the genera that could explain the differences in the gut microbiota of the bird species, we used the indicator command in the MOTHUR program (version v. 1.37.4.).

### Waterbird Phylogeny and Hierarchical Tree of Bacterial Species

To present the taxonomy of the waterbird species, a tree was constructed based on the consensus avian phylogenetic tool available at http://www.birdtree.org (Jetz et al., 2012). The reciprocal tree representing divergences among the different bird species in the gut microbiome was calculated based on Bray–Curtis dissimilarities between communities. MOTHUR tree.shared was used to build a hierarchical tree of species based on Bray–Curtis dissimilarities between samples. The input files for the command were the shared file (i.e., the OTU table) and a design file in which each sample was assigned to bird species.

### Robinson–Foulds and Matching Cluster Congruency Analyses

Congruencies between host phylogenies and intestine microbiota dendrograms were quantified by calculating normalized Robinson–Foulds (RF) (Robinson and Foulds, 1981) and normalized Matching Cluster (MC) metrics (Bogdanowicz and Giaro, 2013), as was described previously (Brooks et al., 2016).

## Results

### Overall Intestine Microbiota Composition

Four different wild waterbird species were collected near fish ponds in northern Israel and three sections of each waterbird intestine were sampled and analyzed. Overall, 87 intestine samples were successfully examined. Bacterial communities’ composition of the intestinal samples were studied using the Illumina MiSeq platform. The filtered, high-quality sequence database obtained was 4,318,583 sequences. These were classified into 40,464 unique operational taxonomic units (OTUs) by a cutoff of 97% sequence similarity. Subsampling according to the smallest sample (20,000 sequences) resulted in 1,560,000 sequences, classified into 33,456 OTUs.

Rarefaction analysis was performed at a threshold of 3% sequences dissimilarity for all samples. The great majority of the samples reached an asymptote level indicating that our sampling efforts were sufficient to obtain an accurate estimate of OTU richness (Supplementary Figure S1). But some did not, and for others the sequencing depth required to reach the asymptote was higher than the sequencing depth for the smallest sample (20,000 sequences). Therefore, although we cannot conclude a census of the microbiota richness, subsampling to the depth of the smallest sample allowed comparison of the bacterial communities’ composition and structure.

**FIGURE 1:**
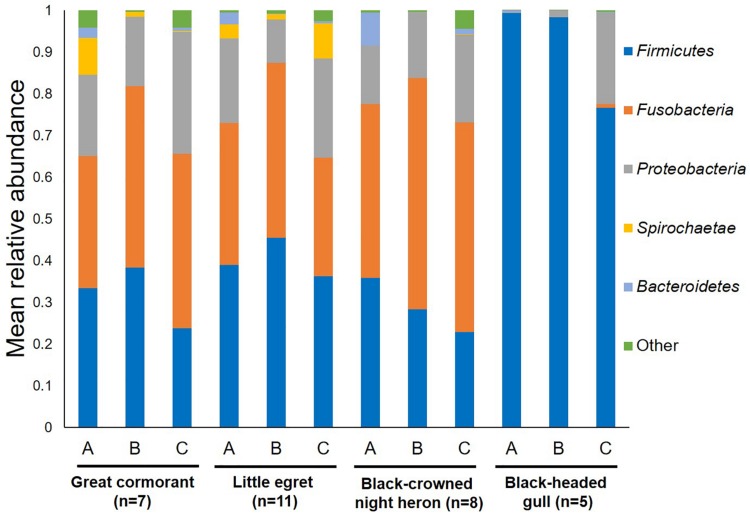
Average OTU abundances at the phyla level. Sum of phyla abundances for each intestine part (A – anterior, B – middle, C – posterior) of all the individual birds belonging to the same species (little egret, black-crowned night heron, great cormorant, black-headed gull). Black-headed gulls differed significantly from all other bird species in *Firmicutes* and *Fusobacteria* (*p* < 0.001). No significant differences were found among all bird species at the phyla level of *Proteobacteria*, *Spirochaetae*, and *Bacteroidetes*.

Overall, 22 different phyla were detected. *Fusobacteria*, *Firmicutes* and *Proteobacteria* were the dominant phyla in all the waterbird samples (Figure 1). *Fusobacteria* was found as the dominant phylum in the great cormorants and black-crowned night herons (39.4 and 48.6% respectively), while *Firmicutes* was dominant in samples from the little egrets and black-headed gulls (40.0 and 90.3%, respectively). In the black-headed gulls the second most dominant phylum was *Proteobacteria* (9.2%). All other phyla were represented by less than 1% of the OTUs (Figure 1). The black-headed gulls differed significantly from all other species in the abundance of *Firmicutes* and *Fusobacteria* (*F*_3__,__27_ = 13.11 and *F*_3__,__27_ = 7.01, respectively, *p* < 0.001). No significant differences were found at the phyla level of *Proteobacteria*, *Spirochaetae* and *Bacteroidetes* among all species (Figure 1).

At the genera level, the most dominant genera identified in the great cormorants were *Fusobacterium*, *Clostridium sensu stricto* and *Campylobacter*, at a prevalence of 32.43, 8.69 and 8.16%, respectively. *Fusobacterium* and *Cetobacterium* were the most dominant genera identified in the little egrets at a prevalence of 19.81 and 10.67%, respectively. The same two genera were also the most dominant in the black-crowned night herons, at a prevalence of 27.30 and 13.33%, respectively. By contrast, the most dominant genera in the black-headed gulls were *Catellicoccus*, *Lactobacillus* and *Clostridium sensu stricto* (58.86, 6.45, and 4.75%, respectively). An unclassified genus belonging to *Clostridiaceae* family was detected at relatively high prevalence (8.18–14.06%) in each of the examined species (Supplementary Table S1).

### The Microbiota of Different Intestinal Sections

When the bacterial community composition in the different intestine sections (anterior, middle, and posterior) were analyzed, all waterbird species except the black-headed gulls shared similar bacterial phyla composition (Figure 1). Nevertheless, the black-headed gulls differed significantly from all other species in *Firmicutes* abundance in each of the intestine sections (A. anterior – *F*_3__,__23_ = 5.71, *p* = 0.005; B. middle – *F*_3__,__23_ = 8.99, *p* = 0.001; C. posterior – *F*_3__,__23_ = 6.59, *p* = 0.002).

ADONIS and Unifrac analyses were applied to assess intestine microbiota variation related to waterbird species, intestinal sections and between-individual variations. Both tests showed similar results and confirmed significant divergence between the microbiota composition of the different species and the effect of individual specimen. However, the different intestinal sections were similar in their microbiota composition (Table 1) (note that because we received similar results for both analyses the data presented is only for ADONIS analysis).

**TABLE 1:** Comparison of waterbird intestine microbiota composition.

**Model**	**df**	***F*_model_**	***R*^2^**	**Pr (>*F*)**
Species	3	4.854	0.169	<0.001
Section	2	0.633	0.014	N.S.
Individual	1	1.654	0.019	0.038
Species: Section	6	0.649	0.045	N.S.
Species: Individual	3	1.452	0.051	0.019
Section: Individual	2	0.721	0.017	N.S.
Species: Section: Individual	6	0.601	0.042	N.S.
Residuals	55		0.641	
Total	78		1	

*Microbiota composition of four waterbird species intestines were compared by ADONIS (10,000 permutations). Waterbird species, intestine section and individual bird-related effects along with all interactions, were examined. df, degrees of freedom; Pr, probability.*

**FIGURE 2:**
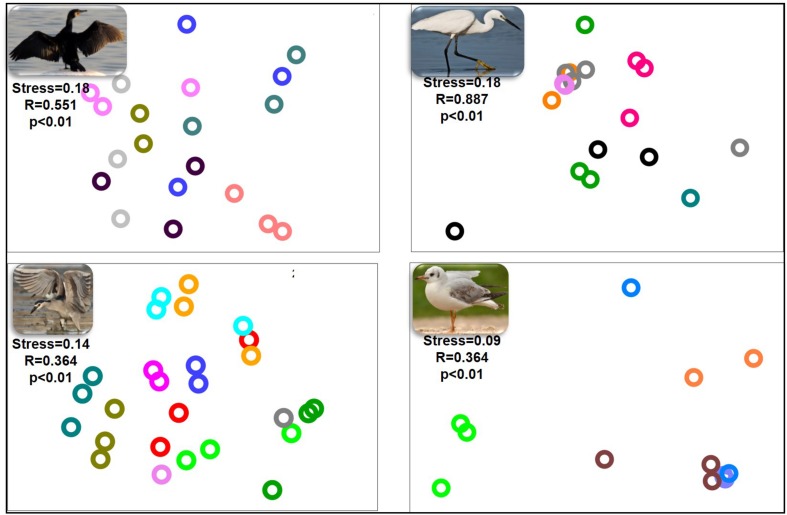
nMDS plot of the intestinal microbiota composition from the different intestinal sections of each individual waterbird. Each part of the figure represents the samples from the intestinal sections of all individuals of the same species. The microbiota composition samples are from great cormorants **(top left)**, black-crowned night herons **(bottom left)**, little egrets **(top right)** and black-headed gulls **(bottom right)**. Each color on each part of the figure represents the three intestinal sections samples from the same individual waterbird (stress value > 0.09). No differences were observed among the microbial communities from the different intestinal sections. Significant differences were observed among the microbial communities of individual birds of the same species. OTUs were determined based on 97% read similarities.

nMDS of the bacterial community composition at the bird species level demonstrated that the microbiota compositions of the three gut sections of an individual bird were alike (ANOSIM *p* > 0.05) (Figure 2). The gut microbiota of the three gut sections of an individual bird were more similar to each other than to the gut microbiota of individual birds of the same species. The dissimilarity levels among individual birds of the four species (little egrets, great cormorants, black-crowned night herons and black-headed gulls) were *R* = 0.887, *R* = 551, *R* = 0.364, and *R* = 0.364, respectively (Figure 2).

### Similarity of Bacterial Communities Among Individuals and Species

We calculated and compared alpha-diversity parameters of the waterbird intestine microbiota composition at the OTU level as well as at the genus level. OTU richness and all genera richness were similar in three species (cormorants, egrets and herons) according to both, Sobs Mean (observed richness) and Chao1 estimator (expected richness). The values of OTU Sobs Mean and OTU Chao1 estimator of the black-headed gulls’ gut microbiota composition were significantly lower, while OTU dominance index was more than twice higher. Similar results were obtained for these indices at the genera level (Table 2). These can be explained by the presence of a specific genus (*Catellicoccus*), with a very high prevalence of more than 50% of the black-headed gulls’ microbiota. All these values suggested that the black-headed gull harbored much lower bacterial community diversity than did the other three bird species. At the OTU and the genera levels, the Sobs mean and the dominance indices showed significant differences among the species (Table 2).

**TABLE 2:** Microbial richness of the four waterbird species (subsampled OTUs at the genera level).

**Phylogenetic level**	**Bird species**	**Great cormorant**	**Little egret**	**Black-crowned night heron**	**Black-headed gull**	**One-way ANOVA**
OTUs	Sobs Mean ± SD	694.6 ± 323.4^a^	595.2 ± 297.4^ab^	618.4 ± 164.7^ab^	227 ± 110^b^	^∗^*F*_3__,__27_ = 3.71 *p* = 0.023
	Chao1 ± SD	889.7 ± 562.9^a^	734.8 ± 337^ab^	778.2 ± 201.1^ab^	307.7 ± 107.5^b^	*F*_3__,__27_ = 2.88 *p* = 0.054
	Dominance ± SD	0.11 ± 0.07^b^	0.101 ± 0.07^b^	0.09 ± 0.05^b^	0.23 ± 0.09^a^	^∗^*F*_3__,__27_ = 5.07 *p* = 0.006
	Evenness ± SD	0.072 ± 0.068^a^	0.071 ± 0.034^a^	0.066 ± 0.041^a^	0.049 ± 0.015^a^	*F*_3__,__27_ = 0.34 *p* = 0.796
All genera	Sobs Mean ± SD	52.8 ± 26.1^a^	50.9 ± 30.2^a^	50.6 ± 48.3^a^	38.7 ± 27.5^a^	*F*_3__,__27_ = 0.19 *p* = 0.902
	Chao1 ± SD	63.6 ± 24.3^a^	73.6 ± 33.1^a^	68.1 ± 48.4^a^	52.8 ± 28.4^a^	*F*_3__,__27_ = 0.41 *p* = 0.744
	Dominance ± SD	0.28 ± 0.13^b^	0.31 ± 0.18^b^	0.3 ± 0.14^b^	0.6 ± 0.27^a^	^∗^*F*_3__,__27_ = 4.05 *p* = 0.017
	Evenness ± SD	0.143 ± 0.078^a^	0.123 ± 0.043^a^	0.135 ± 0.042^a^	0.076 ± 0.029^a^	*F*_3__,__27_ = 1.91 *p* = 0.152

*The Sobs Mean, Chao1, Dominance and Evenness indices were calculated with Past software (version 3.16). Sobs Mean was calculated as the average number of all taxonomic units. Chao1 was calculated as the expected taxonomic richness for the entire collection of each species. Dominance was calculated as the equal of the proportion of the most abundant individual in the sample. Evenness was calculated as the distribution of species’ relative abundances. Statistical analysis was performed by the one-way ANOVA. Asterisk indicates significant differences (*p* < 0.05) among the bird species. Different letters within each row indicate significant differences (*p* < 0.05) among bird species according to *post-hoc* Bonferroni multiple comparison test.*

**FIGURE 3:**
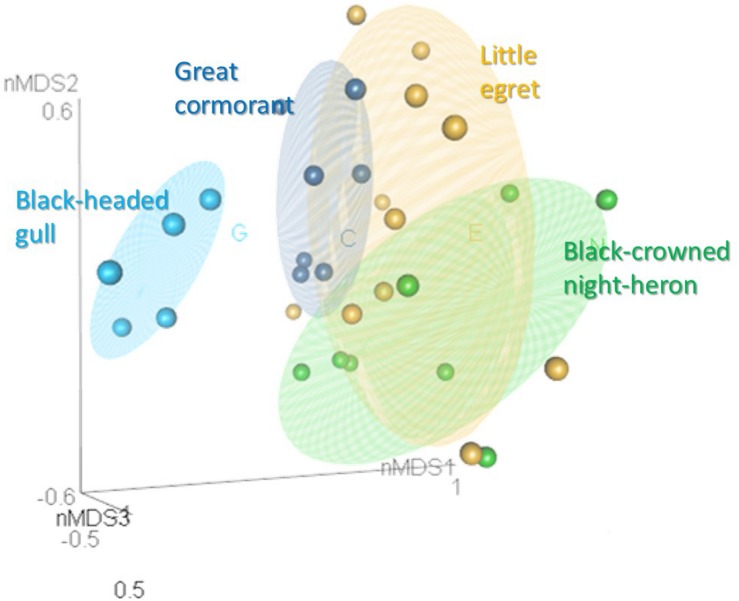
nMDS plot of the intestine bacterial community composition of the waterbird species (stress value = 0.2). Samples from the different intestine sections of each individual bird were combined. OTUs were determined based on 97% read similarities. Bacterial communities of individuals from the same species were clustered together, while significant differences were found between the microbiota of the different species (ANOSIM; *R* = 0.501, *p* = 0.01). More details can be found in Supplementary Table S2.

When the intestine microbiota communities of all the waterbird species were analyzed together by nMDS (Figure 3), the results revealed that individuals of the same species clustered together (*R*^2^ = 0.2). This suggests that each species harbored a unique intestine bacterial community composition (Figure 3). Analysis of similarity (ANOSIM) confirmed the significant differences in the bacterial community composition for each waterbird species (*R* = 0.501, *p* < 0.05) (Supplementary Table S2). Also, ANOSIM showed that each species had a unique bacterial community composition (*p* < 0.05) and that the black-headed gulls’ bacterial community composition showed the greatest differences out of all four bird species (*R* > 0.8). The widest difference was between the black-headed gulls and the great cormorants (*R* = 0.982). Narrower differences were found between the little egrets and the great cormorants and the black-crowned night herons (*R* = 0.229 and *R* = 0.263, respectively) (Supplementary Table S2).

The Venn diagram (Supplementary Figure S2) presents the unique and shared OTUs among the studied samples. For a comparison of the different species, all the intestine sections from all the individual birds of the same species were pooled. Little egrets shared a total of 1,430 unique OTUs, with relative abundance of 65%, with the black-crowned night herons, and 872 unique OTUs, with relative abundance of 60%, with the great cormorants. All four waterbird species shared only 126 unique OTUs (with 26% relative abundance) (Supplementary Figure S2). The Venn diagram of the different sections of the intestine for each bird species showed that the black-headed gulls shared 109 OTUs in all intestine sections with a relative abundance of 85%, the little egrets and the black-crowned night herons shared 299 and 150 OTUs, respectively in all sections and with relative abundances of 68 and 63%, respectively. The great cormorants shared 139 OTUs in all intestine sections and with a very low relative abundance of 1% (Supplementary Figure S3).

We analyzed the presence of genera that had the potential to include pathogenic species in the waterbirds’ intestines. Three potential pathogenic genera were found to dominate the different species: (i) *Fusobacterium*, with high prevalence of 19.81–32.43% in all species except the black-headed gulls; (ii) *Helicobacter*, which was saliently dominant in the black-crowned night herons (11.10%) and the little egrets (7.20%) and (iii) *Clostridium sensu stricto 1*, with occurrence of 4.75–8.69% in all species (Supplementary Table S3). *Vibrio* and *Aeromonas* were detected at low prevalence.

**FIGURE 4:**
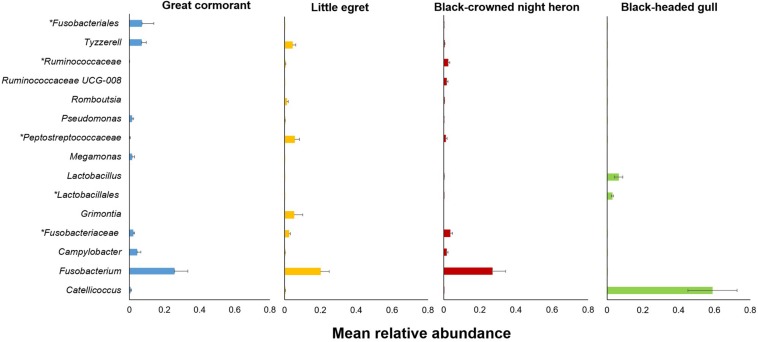
Relative abundances of the 15 most salient genera that contributed to the differences among the four studied waterbird species (Supplementary Table S4). Bars indicate means ± SE. Asterisk indicates a higher taxonomic level.

The genera that contributed most noticeably to the differences among the four waterbird species (indicator genera) are listed in Supplementary Table S4 and in Figure 4. These genera were significantly associated with a specific waterbird species and were found in all intestinal sections of the individual birds of the same species.

### Intestinal Microbiota Heirarchical Tree vs. Birds’ Phylogeny

**FIGURE 5:**
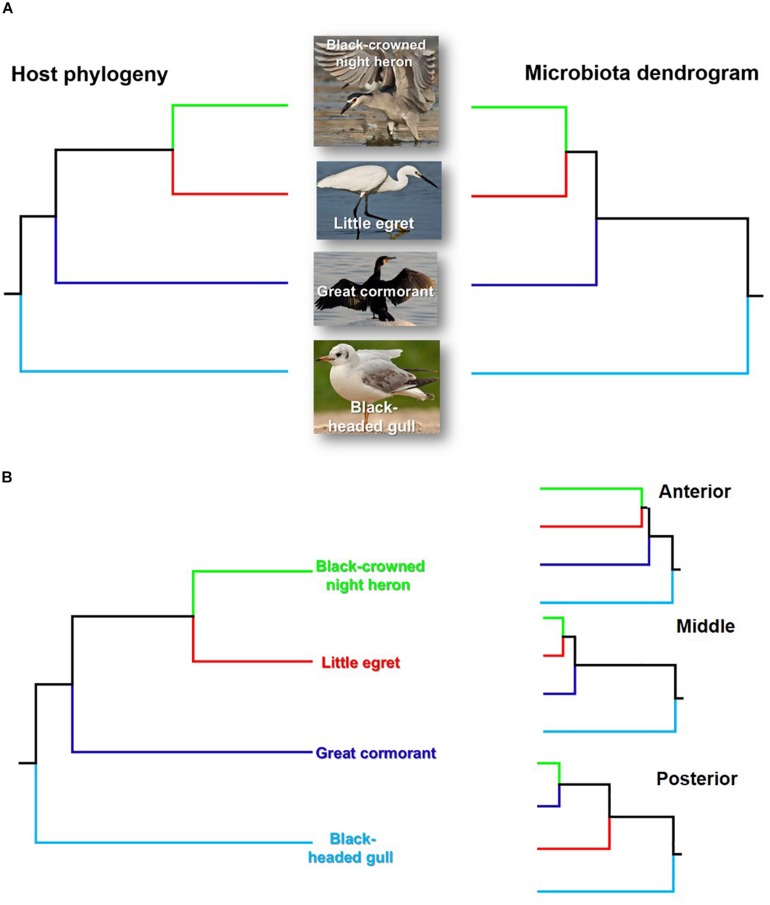
Comparison of the host phylogeny and their microbial community composition. **(A)** Waterbird species phylogeny and their intestine microbiota composition. **(B)** Waterbird species phylogeny vs. the microbiota composition of the three intestine sections (A – anterior, B – middle, C – posterior). The waterbirds phylogenetic tree was assembled using http://www.birdtree.org (Jetz et al., 2012). The intestines’ microbiota dendrogram was constructed using the MOTHUR program (version v. 1.37.4.). The figure indicates that a phylosymbiosis pattern may occur between the waterbird hosts and their intestine microbiota.

The taxonomic tree of the studied waterbirds and the microbiota dendrogram of their intestinal microbiota composition presented a mirror image (Figure 5A), attesting to a correlation between the waterbirds’ phylogeny and their intestine microbiota. This was also found in two out of three gut sections (Figure 5B). To examine whether the waterbirds phylogeny and their intestinal bacterial communities followed patterns of phylosymbiosis, we used the Robinson–Foulds and Matching cluster metrics. Significant patterns of phylosymbiosis were found for the combined data of all the three intestine sections and across two intestine regions (anterior and middle). The posterior intestine region did not demonstrate a significant pattern of phylosymbiosis (Figure 5 and Table 3).

**TABLE 3:** Phylosymbiosis analysis of gut regions.

	**Robinson–Foulds**		**Matching cluster**	
	**nRF**	***p***	**nMC**	***p***
All region	0.0	0.045	0.0	0.045
A. anterior	0.0	0.045	0.0	0.045
B. middle	0.0	0.045	0.0	0.045
C. posterior	0.5	0.314	0.333	0.164

*Normalized Robinson–Foulds (nRF) and normalized Matching Cluster (nMC) scores were determined according to Brooks et al. (2016). Normalized scores scale are from 0.0 (complete congruence) to 1.0 (complete incongruence).*

## Discussion

In this study we examined and compared the microbial community composition of three intestine parts in four wild waterbird species. The nMDS analyses plot of the gut microbiota composition from the different intestinal sections of each individual waterbird, revealed that the gut microbiota of each individual bird of each species hosts a unique microbial community composition (Figure 2). Nevertheless, the nMDS analyses of the intestine bacterial community composition of the four waterbird species demonstrated that each bird species inhabits a unique microbiota composition (Figure 3). Moreover, the results suggest that the intestine microbiota hierarchical tree is consistent with the phylogeny of its waterbirds’ host, providing evidence for phylosymbiosis (Figure 5 and Table 3).

In the current study we sampled the intestinal microbiota composition of waterbirds. As far as we know, most studies of birds’ microbiota used fecal or cloacal samples. However, according to Ingala et al. (2018) and Videvall et al. (2018) these different sampling methods give completely different results and thus are not interchangeable. Videvall et al. (2018) compared the microbiome of cloacal swabs, fecal samples and three different parts of the intestine (ileum, cecum, and colon) of juvenile ostriches (*Struthio camelusfecal*). They concluded that fecal or colon microbiomes are significantly different from the ileum or the cecum microbiomes. Ingala et al. (2018) who compared microbiome sampling methods in wild bats concluded that the microbiomes of fecal and intestine samples differed significantly. While the intestine microbiome probably represents the host-adapted core stable bacterial community, the fecal microbiome represents the food microbiome that is not the host adapted bacterial community but rather passing bacterial communities. The results of both these studies demonstrate that fecal and intestinal sampling methods are not identical, and as such, they provide completely different information (Ingala et al., 2018; Videvall et al., 2018).

*Fusobacteria* and *Firmicutes* were found to be the dominant phyla in the microbial community composition of the intestine samples of the great cormorants, the black-crowned night herons and the little egrets (Figure 1). The third dominant phylum was *Proteobacteria*. Nevertheless, the black-headed gulls differed from all other bird species, with 90% OTUs identified as *Firmicutes* and 9% as *Proteobacteria*, while only 0.3% reads belonged to *Fusobacteria* (Figure 1). The dominant phyla found in poultry (chickens and turkeys) were *Firmicutes*, *Bacteroidetes* and *Proteobacteria*, accounting for more than 90% of all the sequences (Wei et al., 2013). The same dominant phyla were also detected in healthy wild mallards (Ganz et al., 2017) and in a meta-analysis of avian gut microbiota (Waite and Taylor, 2014). A core microbial community composition of *Proteobacteria*, *Firmicutes*, *Bacteroidetes* and *Actinobacteria* was observed in the feces of 59 neotropical bird species (Hird et al., 2015). Similar results were obtained for the microbiomes of the bar-headed goose (*Anser indicus*) (Wang et al., 2016). Kropáčková et al. (2017) found that the dominant phyla in the feces of 51 passerine species were *Proteobacteria*, *Firmicutes* and *Actinobacteria*. *Bacteroidetes* and *Actinobacteria*, detected as dominant phyla in other studies, showed relatively low abundance in ours. By contrast, in the current study, *Fusobacteria* was one of the most abundant phyla (>34%) in all waterbird species except the black-headed gulls (Figure 1). This phylum was not dominant in other avian studies. However, it is noteworthy that most studies were conducted on fecal samples, while in the current study, samples were taken directly from the intestine and as such represent the host adapted bacterial communities.

Interestingly, one genus, *Catellicoccus*, was dominant in the black-headed gulls, with an average relative abundance of 58.86% (Figure 4 and Supplementary Table S4). *Catellicoccus marimammalium* belongs to the *Enterococcaceae* family, and is a facultative anaerobic, Gram-positive species. It was originally isolated from the carcasses of a porpoise and a gray seal (Lawson et al., 2006). *Catellicoccus* was found dominant in a variety of gulls’ fecal samples (Ryu et al., 2012; Sinigalliano et al., 2013; Koskey et al., 2014), suggesting that it could be a symbiont and or associated with their particular diets and lifestyle (Figure 4 and Supplementary Table S4).

nMDS analyses revealed that the gut microbiota of each individual bird of each species, hosted a unique microbial community composition (Figures 2, 3). Differences among individuals can be explained by the diet variation among individuals of each waterbird species. Many studies showed that individual specialization in diet is widespread among generalist predators (e.g., Bolnick et al., 2003). The variation in diet among individuals within the population is a consequence of their specialized foraging behavior, sex, age, health condition, body size, experience and dominance (Field et al., 2007; Woo et al., 2008). Another explanation is the predator-prey theory. According to this theory, it is expected that there may be differences between individual birds of the same species due to individual differences in their diet, but larger differences among different species, especially those with less diet overlap. Indeed, the comparison of all individuals of each waterbird species showed that each harbored a unique intestine microbiota (ANOSIM, *R* = 0.501) (Figure 3). Although there is some overlap among the diets of the four species, each one of them has distinct dietary items. The great cormorants catch their prey by pursue diving. In Lake Kinneret in northern Israel they feed on 10 fish species, mainly *Acanthobrama terraesanctae* (27% of the analyzed stomachs, *n* = 77 birds), *Sarotherodon galilaeus* (19%) and *Tilapia zillii* (18%) (Izhaki, unpublished data). The black headed gulls is an opportunist whose diet consists of invertebrates, small vertebrates, small fish and waste from municipal landfills and farms (Čížek et al., 2007). The diet, feeding techniques and foraging behavior of the two closely related species in our study (the herons) are rather distinct. Little egret is a diurnal forager, usually forage in open habitats, a fast runner and chaser, and mainly consumes small fish and insects, whereas night heron is nocturnal, usually forage in dense vegetation habitats, a relatively slow and not a chaser and mainly consumes amphibians and medium sized fish (Ashkenazi and Dimentman, 1998; Katzir et al., 2001). A previous study in aquaculture fish farms in northern Israel (Ashkenazi and Yom-Tov, 2006) showed that little egrets’ fish diet contained 50.9% tilapia and 38.3% carp whereas night herons’ fish diet contained 82.7% tilapia and only 6.5% carp. Moreover, little egrets consume much smaller tilapia fish than night herons (3.2 ± 2.8 g, *n* = 799 consumed fish and 23.4 g ± 17.6, *n* = 219, respectively). Furthermore, 14.9% (*n* = 84 birds) of little egrets’ stomach samples contained aquatic insects (*Corixidae, Notonectidae*), while only 1.3% (*n* = 207 birds) of night herons’ stomach samples contained these insects (Ashkenazi and Yom-Tov, 2006). The nMDS ordination of the microbiomes reflects these diet similarities and dissimilarities among the four waterbird species. The individual black-headed gulls clustered together, and apart from the other waterbird species. The two herons were clustered relatively close, whereas the cormorants were clustered relatively close to each other with some overlap with the little egrets (Figure 3).

We studies wild waterbird species. Microbiomes of wild animals have been studied mainly in terrestrial mammals. Birds account for more than 15% of vertebrates, but most studies have focused on terrestrial birds – mainly poultry (Pearce et al., 2017). Dietary, social and environmental conditions in animals kept in captivity are different from those of wild animals (Hird, 2017) and might make a difference in microbiota composition.

Some genera identified from the waterbird intestines were potentially human pathogens. For example, *Clostridium sensu stricto 1* (which includes *C. tetani, C. botulinum, C. kluyveri, C. acetobutylicum, C. novyi, C. perfringens* and *C. beijerinckii*) (Supplementary Table S3). *Clostridium sensu stricto 1* was found in all the waterbird species and at relatively high percentages (above 4.75%). *Clostridium* species may cause tetanus, botulism, etc. (Bruggemann et al., 2003; Twine et al., 2008). *Fusobacterium* (Supplementary Table S3) includes species like *F. necrophorum* which can cause human oral infections, and *F. nucleatum* which can cause inflammatory bowel disease (Tan et al., 1996; Allen-Vercoe et al., 2011). Other genera with pathogenic species or potential pathogenic genera were found in some waterbird species, for example, *Aeromonas*, *Clostridium, Helicobacter*, *Campylobacter, Arcobacter*, *Vibrio*, etc. (Supplementary Table S3). Halpern et al. (2008), suggested that waterbirds can potentially transfer *V. cholerae* globally and this may be the way new endemic foci of cholera come to be established remote from the source of the infection. Likewise, waterbirds migration across national and intercontinental borders provides a mechanism for global dispersion of bacterial species, including other pathogen species. Ryu et al. (2014) who studied migratory shorebirds also pointed at the fact that they may be potential reservoirs of pathogenic *Campylobacter* species and also of other pathogenic species. However, it has to be clarified that the presence of OTUs that belong to these potentially human pathogens does not necessarily mean that these OTUs are pathogenic species. In the current study, we did not study the presence of pathogenic islands of potentially pathogenic species, thus, complementary research using either culturable methods or metagenomics should be performed to verify this hypothesis.

The current study suggests that the waterbirds’ phylogeny and their intestine bacterial community composition hierarchical tree are congruent (Figure 5A). Similar patterns were also observed for the birds’ taxonomy and the intestine microbiota composition of the three intestine sections (Figure 5B). This matching pattern of the host’s phylogeny and its intestine microbiota composition is termed phylosymbiosis (Brucker and Bordenstein, 2012a, b; Theis et al., 2016) and is considered to be a result of eco-evolutionary processes. Although Brooks et al. (2016) pointed out that phylosymbiosis may occur only under controlled environmental conditions, here we were able to demonstrate that phylosymbiosis patterns can be observed also in wild waterbird species that we had no control on the environments they visited, or on the food items that they consumed.

Little egrets and black-crowned night herons are relatively closely related as they belong to the same family (*Ardeidae*) (Shirihai et al., 1996). Accordingly, their gut microbiota communities presented relatively similar patterns. Great cormorants and black-headed gulls were more distinct from egrets and herons, as was their gut microbiota composition (Figure 5A). Cormorants belong to a different family but to the same order as little egrets and black-crowned night herons (*Pelecaniformes*) (Jarvis et al., 2014). Gulls and their gut bacterial communities are situated in the tree as an outgroup, probably since they belong to a different order (*Charadriiformes*) (Hackett et al., 2008) (Figure 5A). However, the parallel patterns of the microbiome and the phylogenetic distances among the four species could emerge due to other ecological differences among the species that correlate with phylogenetic distances such as diet. Thus, it should be emphasized that ecological differences associated with diversification can impact microbial community composition, independently of direct co-evolutionary responses. Therefore, other ecological factors rather than only phylosymbiosis can shape the mirror image of the phylogentic tree and the microbiome dendogram which we found here (Figure 5A).

Patterns of phylosymbiosis have previously been observed in *Peromyscus*, deer mice, *Drosophila* flies, mosquitoes (*Anopheles*/*Aedes*/*Culex*), *Nasonia* wasps, wild hominids (Brooks et al., 2016); American pikas (*Ochotona princeps*) (Kohl et al., 2017) and different coral reef fish species (Chiarello et al., 2018). However, phylosimbiosis in birds has rarely been described. Kropáčková et al. (2017) studied the microbial composition of 51 passerine species. They showed closer correlation between gut microbiome divergence and the hosts’ phylogenetic divergence than between the gut microbiome and the ecological or life-history traits and geographic variations. These results suggest that a phylosymbiosis pattern may exists between the passerine species’ phylogeny and their microbiomes; however, the authors did not suggest that this phenomenon may be relevant to their study (Kropáčková et al., 2017).

## Conclusion

Here we compared the gut bacterial community composition of four wild waterbird species. Our results demonstrate that although individual birds from each bird species differed, each species inhabited a unique gut bacterial community composition. Potential pathogenic genera were identified in the gut microbiota of the different species, suggesting that waterbirds may disseminate pathogenic species like *Clostridium, Helicobacter*, *Campylobacter, Vibrio*, etc., between waterbodies. Moreover, our results suggest that the gut microbiota composition is consistent with the phylogeny of its waterbirds’ host, providing evidence for phylosymbiosis. Although Brooks et al. (2016) suggested studying the phylosymbiosis theory under laboratory controlled experiments to find more evidence regarding this theory, we were able to show this pattern in wild waterbird species. We conclude that eco-evolutionary processes may occur between wild waterbird species and their gut bacterial community composition, providing evidence for the holobiont eco-evolutionary process theory.

## Data Availability

The datasets generated for this study can be found in NCBI, BioProject accession number PRJNA336254.

## Author Contributions

SL-S, II, and MH conceived and designed the experiments. SL-S performed the experiments. II and MH contributed to reagents, materials, and analysis tools. SL-S and ML analyzed the data. SL-S and MH wrote the manuscript. All authors discussed the results, and reviewed and commented the manuscript.

## Conflict of Interest Statement

The authors declare that the research was conducted in the absence of any commercial or financial relationships that could be construed as a potential conflict of interest.

## Footnotes

1 www.unep-aewa.org

2 https://www.ncbi.nlm.nih.gov/sra
