# Supplementary material for: Comparative Analysis of Intestine Microbiota of Four Wild Waterbird Species
Source: Front Microbiol. 2019 Aug 20;10:1911. doi: 10.3389/fmicb.2019.01911 (PMC6711360; doi:10.3389/fmicb.2019.01911)
Supplement: Supplementary file 1 [file Data_Sheet_1.pdf]

Comparative Analysis of Intestine Microbiota of Four Wild Waterbird Species

Sivan Laviad-Shitrit, Ido Izhaki, Maya Lalzar and Malka Halpern

Supplementary files

Supplementary Tables

Table S1. A list of the genera and their abundances within each waterbird intestine sample (C; great cormorant, E; little egret, B; black-headed gull, N; and black-crowned night heron) and intestine sections (A - anterior, B - middle and C - posterior). Table S1 is attached in a separate Excel file.

Table S2. **ANOSIM analysis among waterbird species**. R statistic represents similarity among the microbial community compositions of the indicated waterbird species. R can vary between 0.0 and 1.0, where 0.0 represents a similar bacterial community composition and 1.0 represents a markedly different bacterial community composition.

Table S3. Potential pathogenic genera identified in the waterbird intestine samples and were present with at least 0.5% prevalence, in at least one of the waterbird species.

Table S4. Genera that contributed most saliently to differences among the four waterbird species (indicator genera). Each indicator genus was detected in all the samples of a specific waterbird species. Indicator value represents fidelity and relative abundance. These indicator genera were found significantly responsible for the differences among the waterbird species $p < 0.05$.

Supplementary Figures

Figure S1. **Rarefaction curves** indicating the observed number of operational taxonomic units (OTUs) at a genetic distance of 3% in all samples. Rarefaction curves display the increase in the number of the OTUs as a function of the sequence numbers of each sample.

Figure S2. Venn diagram representing the number of unique and shared OTUs (97% sequences similarity) between the different waterbird species. All intestine sections from all birds belonging to the same waterbird species were combined. The relative abundances of the OTUs are indicated by precentages.

Figure S3. Venn diagram representing the number of unique and shared OTUs (97% sequence similarity) between the diffrenet intestine sections (A – anterior; B – middle; C – posterior) that were studied for each waterbird species. The relative abundance of the OTUs is indicated by precentages.

Supplementary Tables

Table S2. **ANOSIM analysis among waterbird species**. R statistic represents similarity among the microbial community compositions of the indicated waterbird species. R can vary between 0.0 and 1.0, where 0.0 represents a similar bacterial community composition and 1.0 represents a markedly different bacterial community composition.

**waterbird species**	**R statistic**	**Significance level**
little egret + great cormorant + black-headed gull + black-crowned night heron	0.501	0.001
little egret + great cormorant	0.229	0.024
great cormorant + black-headed gull	0.982	0.001
great cormorant + black-crowned night heron	0.316	0.002
little egret + black-headed gull	0.841	0.003
little egret + black-crowned night heron	0.263	0.007
black-headed gull + black-crowned night heron	0.829	0.002

Table S3. Potential pathogenic genera identified in the waterbird intestine samples and were present with at least 0.5% prevalence, in at least one of the waterbird species.
Bold numbers represent the number of birds positive for the bacterial genus out of the total number of the birds that were examined from the same bird species. Percentages in parentheses represent the relative abundance of the bacterial genus in the intestine of individuals that harbored this genus. References on pathogenicity potential are indicated.

Clostridium sensu stricto 1 includes the following species: *C. tetani*, *C. botulinum*, *C. kluyveri*, *C. acetobutylicum*, *C. novyi*, *C. perfringens* and *C. beijerinckii*.

	**Great cormorant**	**Little egret**	**Black-crowned night heron**	**Black-headed gull**	**Reference**
Bacteroidetes					
Bacteroides	**4/7** (0.003%)	**6/11** (0.61%)	**3/8** (0.47%)	**0/5**	(Sears et al., 2008)
Firmicutes					
Clostridium sensu stricto 1	**7/7** (**8.69%**)	**11/11 (7.27%)**	**8/8** (4.84%)	**4/5** (**5.93%**)	(Bruggemann et al., 2003; Twine et al., 2008)
Staphylococcus	**6/7** (0.23%)	**6/11** (0.21%)	**3/8** (0.31%)	**4/5** (0.03%)	(Oliveira et al., 2002)
Streptococcus	**7/7** (0.18%)	**9/11** (0.07)	**4/8** (0.14%)	**5/5** (0.05%)	(Lun et al., 2007)
Fusobacterium	**7/7 (32.43%)**	**11/11 (19.81%)**	**8/8** (**27.30%**)	**4/5** (0.008%)	(Allen-Vercoe et al., 2011; Tan et al., 1996)
Proteobacteria					
Aeromonas	**5/7** (0.19%)	**11/11** (0.26%)	**4/8** (0.70%)	**5/5** (0.67%)	(Laviad and Halpern, 2016)
Arcobacter	**2/7** (0.11%)	**1/11** (0.003%)	**1/8** (0.1%)	**0/5**	(Vandenberg et al., 2004)
Campylobacter	**7/7 (8.01%)**	**10/11** (0.28%)	**7/8** (1.68%)	**3/5** (0.01)	(Wassenaar and Newell, 2006)
Escherichia-Shigella	**6/7**(0.04%)	**10/11** (0.57%)	**6/8** (0.01%)	**4/5** (3.01%)	(Croxen and Finlay, 2010)
Halomonas	**7/7** (0.49%)	**7/11** (0.18%)	**3/8** (0.07%)	**4/5** (0.016%)	(Stevens et al., 2009)
Helicobacter	**7/7** (0.91%)	**10/11(7.92%)**	**8/8** (11.10%)	**3/5** (0.07%)	(Ertem, 2013)
Paracoccus	**5/7** (0.20%)	**8/11** (0.04%)	**4/8** (0.12%)	**4/5** (2.16%)	(Daneshvar et al., 2003)
Plesiomonas	**6/7**(0.09%)	**9/11** (0.09%)	**5/8** (0.04%)	**2/5** (0.01%)	(Brenden et al., 1988)
Pseudomonas	**7/7** (1.54%)	**11/11** (0.31%)	**7/8** (0.07%)	**5/5** (0.05%)	(Mena and Gerba, 2009)
Psychrobacter	**1/7** (0.22%)	**2/11** (0.009%)	**2/8** (0.35%)	**1/5** (0.001%)	(Gini, 1990)
Sphingomonas	**5/7** (0.12%)	**8/11**(0.02%)	**1/8** (0.17%)	**3/5** (0.005%)	(Lin et al., 2010)
Stenotrophomonas	**4/7** (0.21%)	**4/11** (0.08%)	**3/8** (0.009%)	**1/5** (0.005%)	(Looney et al., 2009)
Vibrio	**5/7**(0.94%)	**7/11** (0.02%)	**7/8** (0.49%)	**4/5** (0.005%)	(Daniels and Shafaie, 2000)

References

Allen-Vercoe, E., Strauss, J., and Chadee, K. (2011). *Fusobacterium nucleatum*: An emerging gut pathogen? *Gut Microbes* 2, 294–298. doi:10.4161/gmic.2.5.18603.

Brenden, R. A., Miller, M. A., and Janda, J. M. (1988). Clinical disease spectrum and pathogenic factors associated with *Plesiomonas shigelloides* infections in humans. *Clin. Infect. Dis.* 10, 303–316. doi:10.1093/clinids/10.2.303.

Bruggemann, H., Baumer, S., Fricke, W. F., Wiezer, A., Liesegang, H., Decker, I., et al. (2003). The genome sequence of *Clostridium tetani*, the causative agent of tetanus disease. *Proc. Natl. Acad. Sci.* 100, 1316–1321. doi:10.1073/pnas.0335853100.

Croxen, M. A., and Finlay, B. B. (2010). Molecular mechanisms of *Escherichia coli* pathogenicity. *Nat. Rev. Microbiol.* 8, 26–38. doi:10.1038/nrmicro2265.

Daneshvar, M. I., Hollis, D. G., Weyant, R. S., Steigerwalt, A. G., Whitney, A. M., Douglas, M. P., et al. (2003). *Paracoccus yeeii* sp. nov. (Formerly CDC group EO-2), a novel bacterial species associated with human infection. *J. Clin. Microbiol.* 41, 1289–1294. doi:10.1128/JCM.41.3.1289-1294.2003.

Daniels, N. a, and Shafaie, A. (2000). A review of pathogenic *Vibrio* infections for clinicians. *Infect. Med.* 17, 665–685.

Ertem, D. (2013). Clinical Practice: *Helicobacter pylori* infection in childhood. *Eur. J. Pediatr.* 172, 1427–1434. doi:10.1007/s00431-012-1823-4.

Gini, G. A. (1990). Ocular infection caused by *Psychrobacter immobilis* acquired in the hospital. *J. Clin. Microbiol.* 28, 400–401.

Laviad, S., and Halpern, M. (2016). Chironomids' relationship with *Aeromonas* species. *Front. Microbiol.* 7. doi:doi: 10.3389/fmicb.2016.00736.

Lin, J. N., Lai, C. H., Chen, Y. H., Lin, H. L., Huang, C. K., Chen, W. F., et al. (2010). *Sphingomonas paucimobilis* bacteremia in humans: 16 case reports and a literature review. *J. Microbiol. Immunol. Infect.* 43, 35–42. doi:10.1016/S1684-1182(10)60005-9.

Looney, W. J., Narita, M., and Mühlemann, K. (2009). *Stenotrophomonas maltophilia*: an emerging opportunist human pathogen. *Lancet Infect. Dis.* 9, 312–323. doi:10.1016/S1473-3099(09)70083-0.

Lun, Z. R., Wang, Q. P., Chen, X. G., Li, A. X., and Zhu, X. Q. (2007). *Streptococcus suis*: an emerging zoonotic pathogen. *Lancet Infect. Dis.* 7, 201–209. doi:10.1016/S1473-3099(07)70001-4.

Mena, K. D., and Gerba, C. P. (2009). Risk assessment of *Pseudomonas aeruginosa* in water. *Rev. Environ. Contam. Toxicol.* 201, 71–115. doi:10.1007/978-1-4419-0032-6_3.

Oliveira, D. C., Tomasz, A., and De Lencastre, H. (2002). Secrets of success of a human pathogen: Molecular evolution of pandemic clones of meticillin-resistant *Staphylococcus aureus*. *Lancet Infect. Dis.* 2, 180–189. doi:10.1016/S1473-3099(02)00227-X.

Sears, C. L., Islam, S., Saha, A., Arjumand, M., Alam, N. H., Faruque, A. S. G., et al. (2008). Association of enterotoxigenic *Bacteroides fragilis* infection with

inflammatory diarrhea . *Clin. Infect. Dis.* 47, 797–803. doi:10.1086/591130 [doi].

Stevens, D. A., Hamilton, J. R., Johnson, N., Kim, K. K., and Lee, J. S. (2009). *Halomonas*, a newly recognized human pathogen causing infections and contamination in a dialysis center: Three new species. *Medicine (Baltimore).* 88, 244–249. doi:10.1097/MD.0b013e3181aede29.

Tan, Z. L., Nagaraja, T. G., and Chengappa, M. M. (1996). *Fusobacterium necrophorum* infections: virulence factors, pathogenic mechanism and control measures. *Vet. Res. Commun.* 20, 113–140. doi:10.1007/BF00385634.

Twine, S. M., Paul, C. J., Vinogradov, E., McNally, D. J., Brisson, J. R., Mullen, J. A., et al. (2008). Flagellar glycosylation in *Clostridium botulinum*. *FEBS J.* 275, 4428–4444. doi:10.1111/j.1742-4658.2008.06589.x.

Vandenberg, O., Dediste, A., Houf, K., Ibekwem, S., Souayah, H., Cadranel, S., et al. (2004). *Arcobacter* species in humans. in *Emerging Infectious Diseases*, 1863–1867. doi:10.3201/eid1010.040241.

Wassenaar, T. M., and Newell, D. G. (2006). “The Genus *Campylobacter*,” in *The Prokaryotes*, 119–138. doi:10.1007/0-387-30747-8_4.

Table S4. Genera that contributed most saliently to differences among the four waterbird species (indicator genera). Each indicator genus was detected in all the samples of a specific waterbird species. Indicator value represents fidelity and relative abundance. These indicator genera were found significantly responsible for the differences among the waterbird species $p < 0.05$. See also Fig. 4.
Asterisk indicates a taxonomic higher level.

**Genera**	**Bird species**	**Indicator value (%)**
**Fusobacteriales*	Great cormorant	35.71%
Tyzzerell	Great cormorant	64.88%
**Ruminococcaceae*	Black-crowned night heron	41.36%
Ruminococcaceae UCG-008	Black-crowned night heron	57.82%
Romboutsia	Little egret	57.53%
Pseudomonas	Great cormorant	67.92%
**Peptostreptococcaceae*	Little egret	66.88%
Megamonas	Great cormorant	84.53%
Lactobacillus	Black-headed gull	97.62%
**Lactobacillales*	Black-headed gull	99.77%
Grimontia	Little egret	40.73%
**Fusobacteriaceae*	Black-crowned night heron	45.63%
Campylobacter	Great cormorant	74.41%
Fusobacterium	Great cormorant	37.45%
Catellicoccus	Black-headed gull	98.33%

Supplementary Figures

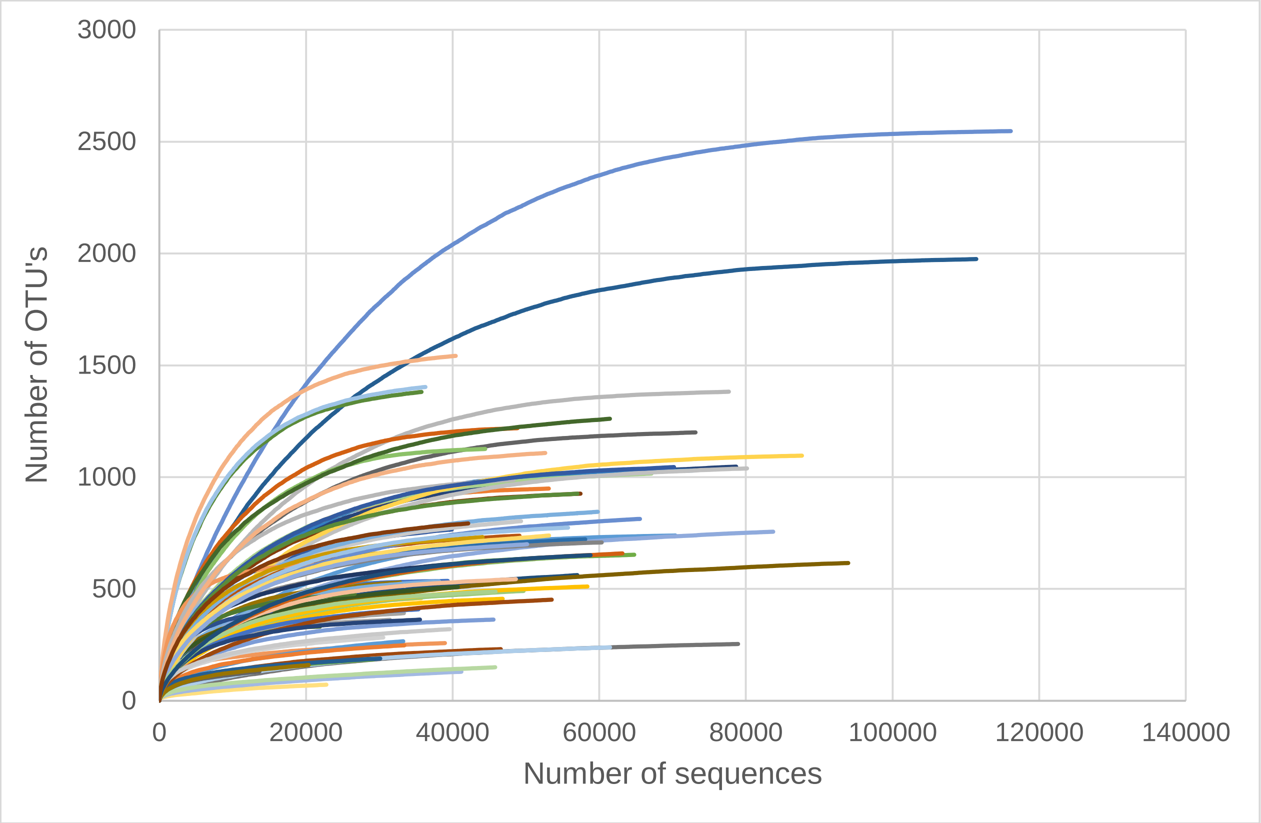

Figure S1. **Rarefaction curves** indicating the observed number of operational taxonomic units (OTUs) at a genetic distance of 3% in all samples. Rarefaction curves display the increase in the number of the OTUs as a function of the sequence numbers of each sample.

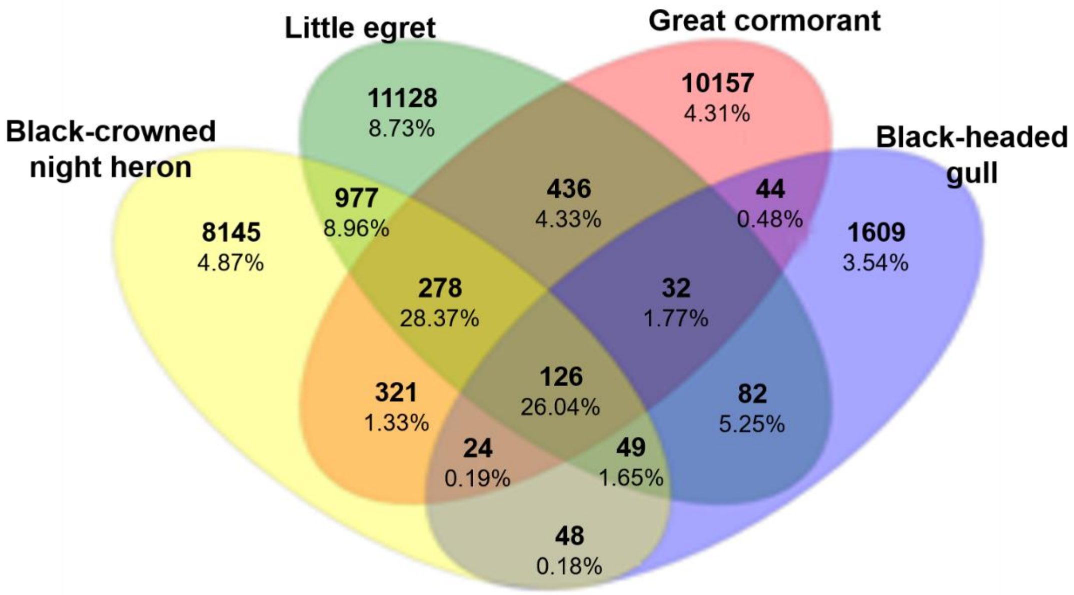

Figure S2. Venn diagram representing the number of unique and shared OTUs (97% sequences similarity) between the different waterbird species. All intestine sections from all birds belonging to the same waterbird species were combined. The relative abundances of the OTUs are indicated by precentages.

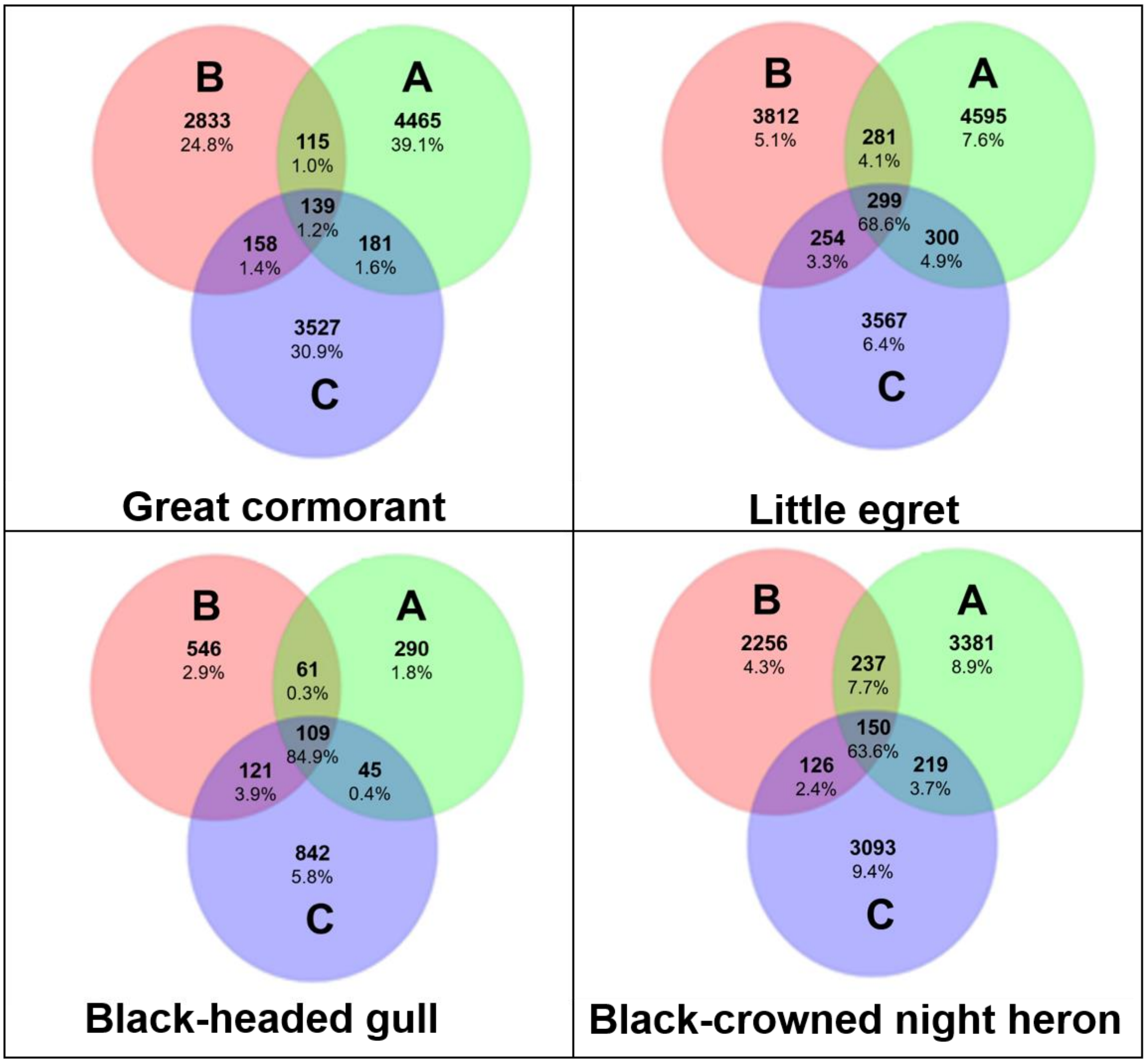

Figure S3. Venn diagram representing the number of unique and shared OTUs (97% sequence similarity) between the diffrenet intestine sections (A – anterior; B – middle; C – posterior) that were studied for each waterbird species. The relative abundance of the OTUs is indicated by precentages.
